# Rebuilding a protective allele with ADAR site-directed RNA editing

**DOI:** 10.1016/j.omtn.2026.103097

**Published:** 2026-09-26

**Authors:** Michael F. Jantsch, Ângela S. Marques

**Affiliations:** 1 Division of Cell and Developmental Biology, Center for Anatomy and Cell Biology, Medical University of Vienna, Vienna, Austria

## Abstract

De Chiara F, Coll-de la Rubia E, de Bruijn P et al. ADAR RNA editing for cardiovascular disease: Targeting B4GALT1 to modulate lipid metabolism through reduced galactosyltransferase activity. Molecular Therapy Nucleic Acids. 2026;37(3):103001. https://doi.org/10.1016/j.omtn.2026.103001, this issue.

## Main text

Site-directed RNA editing (SDRE) is an emerging class of RNA-based therapeutics that recodes a single nucleotide within a chosen messenger RNA without altering the genome.^1^ A short chemically modified oligonucleotide hybridizes to the target transcript, creating a defined A•C mismatch that recruits an endogenous adenosine deaminase acting on RNA (ADAR). ADAR converts the targeted adenosine (A) to inosine (I), which the ribosome reads as guanosine, thus yielding a defined amino acid substitution. Because no exogenous editing enzyme, viral vector, or permanent DNA change is introduced, the edit persists only as long as the oligonucleotide is dosed, making the approach titratable and reversible—unlike genomic base editing or RNA-degrading strategies such as those based on small interfering RNAs (siRNAs) and antisense gapmers–and, therefore, safer.

Lowering LDL cholesterol has been a great success in cardiovascular medicine, yet the available approaches differ in efficacy and side effects.^2^ An alternative to asking which pathways can be blocked is to ask which populations are naturally protected, and why. In this issue of Molecular Therapy—Nucleic Acids, De Chiara and colleagues do exactly that^3^: they use chemically modified editing oligonucleotides (EONs) to recruit endogenous ADAR and introduce the cardioprotective Amish variant p.Asn352Ser into hepatic *B4galt1* mRNA in mice. An AAC or AAU “asparagine” codon is, thereby, converted into AIC or AIU, which is read as “serine.”

Editing of fewer than 20% of transcripts reduced hepatic galactosyltransferase activity by ∼30%, total cholesterol by ∼60%, and ApoB by ∼70% in Western diet-fed APOE∗3-Leiden.CETP mice, while *B4galt1* mRNA and protein levels remained unchanged.

## Protective alleles as therapeutic aims

Human genetics is a reliable source of cardiovascular target discovery: loss-of-function variants in *PCSK9*, *ANGPTL3*, and *LPA* identify carriers with low lipids and low event rates,^4^ and each has been translated into a drug.^2^ Such interventions almost always aim for ablating the protein with an antibody, siRNA, or antisense oligonucleotide. This strategy, however, works only when the protein is dispensable, which B4GALT1 is not. B4GALT1 catalyzes terminal β1,4-galactosylation of N-glycans on secreted hepatic proteins such as ApoB,^5^ and the resulting glycan architecture governs how fast those proteins are cleared.^6^ Its p.Asn352Ser variant, enriched in the Old Order Amish population, halves the galactosyltransferase activity and lowers LDL-C and fibrinogen, thus reducing cardiovascular risk in otherwise healthy carriers^5^ (Figure 1A). Complete loss of B4GALT1, by contrast, causes a congenital disorder of glycosylation with hepatointestinal and coagulation abnormalities,^7^ and knockout mice show delayed growth and early death.^8^ The therapeutic aim is, therefore, not to inactivate the enzyme but to reduce its activity—a dose-controlled task for which SDRE is well suited (Figure 1B), and one in which limited editing efficiency becomes a dosing dial, rather than a limitation.

**Figure 1:**
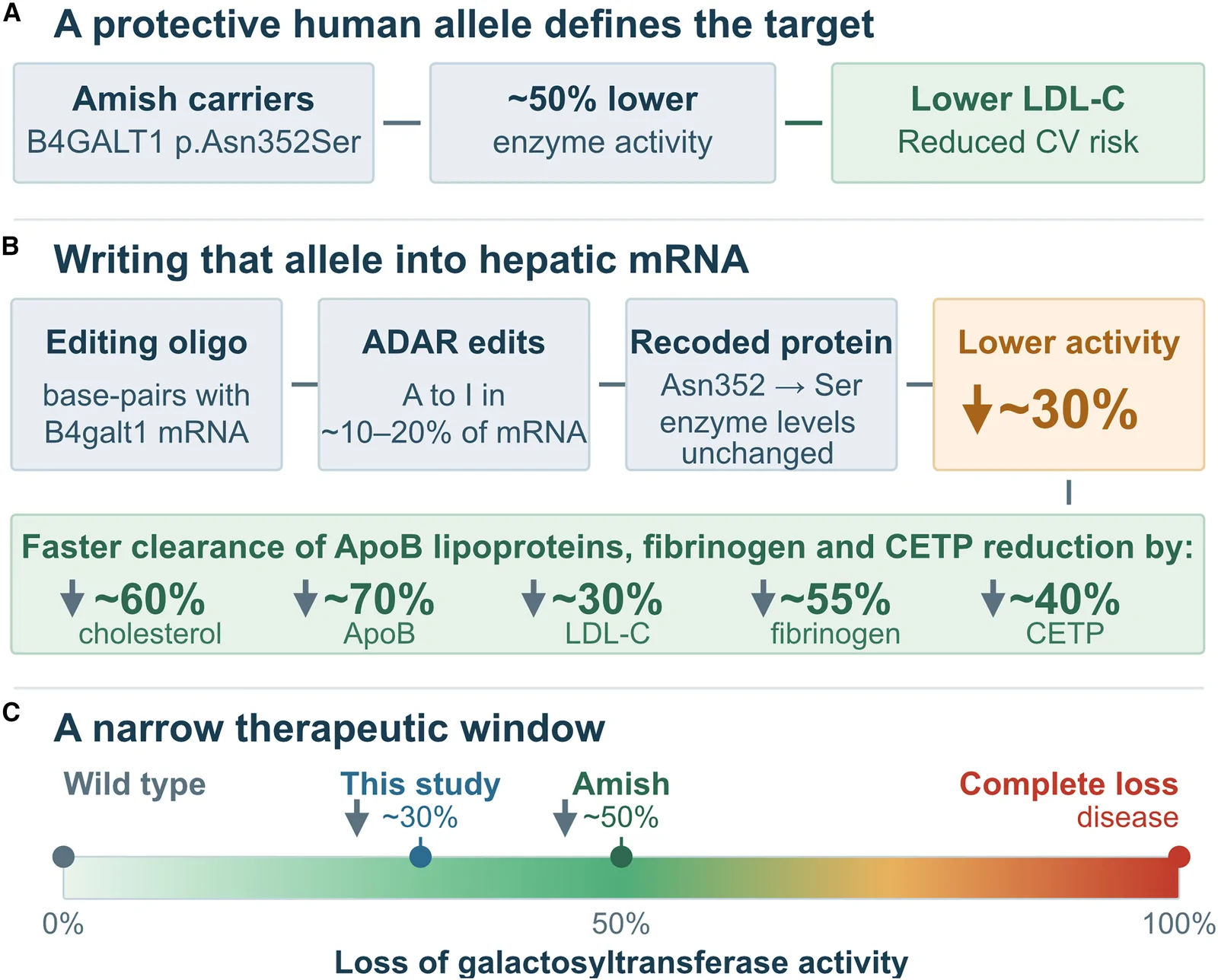
Copying a cardioprotective human allele into hepatic mRNA (A) The p.Asn352Ser variant of B4GALT1, enriched in the Old Order Amish, roughly halves the galactosyltransferase activity without abolishing the enzyme and is associated with lower LDL-C and fibrinogen and reduced cardiovascular risk. (B) An editing oligonucleotide delivered to the liver base-pairs with *B4galt1* mRNA and recruits endogenous ADAR, which deaminates the target adenosine to inosine; the ribosome reads inosine as guanosine, substituting Asn352 for Ser. Transcript and protein abundances are unchanged, but galactosyltransferase activity falls by ∼30%; plasma glycoproteins lose terminal galactose, sialic acid, and core fucose; and ApoB-containing lipoproteins, fibrinogen, and cholesteryl ester transfer protein (CETP) are cleared faster. (C) Human genetics brackets the therapeutic window from both sides: about half of normal activity is protective, while complete deficiency causes a congenital disorder of glycosylation in humans and growth delay with early death in mice. The ∼30% activity reduction achieved here sits inside the protective window and derives from editing only 9%–18% of transcripts—an amplification that follows from editing a catalytic enzyme.

## What the study shows?

In wild-type mice, a first-generation oligonucleotide (EON-1) reached ∼6% editing with hepatic exposure peaking at 24 h, leaving *B4galt1* transcript and protein abundance unaffected. A chemically improved EON-2 was then dosed weekly for 45 days in Western diet-fed APOE∗3-Leiden.CETP mice, a model widely used to test lipid-lowering agents. Editing of ∼15% was sustained throughout, with no bystander editing detectable by deep sequencing. Critically, hepatic galactosyltransferase activity fell by ∼30%, while enzyme abundance did not—direct evidence that recoding, not knockdown, drove the phenotype. Plasma glycomics showed coordinated reductions in galactosylation, sialylation, and core fucosylation, recapitulating the glycan signature of human carriers of hypomorphic *B4GALT1* alleles. Longitudinal plasma proteomics resolved three trajectories: sustained lipid suppression, dynamic coagulation and complement regulation, and a transient inflammatory signature that resolved by day 45.

## Open questions and perspectives

ADAR editing crossed into the clinic toward the end of 2023 (Table 1)^9^; N-acetylgalactosamine (GalNAc)-delivered WVE-006 restored a less severe phenotype in patients with α1-antitrypsin deficiency (AATD), and the same Axiomer chemistry used here was applied by ProQR to produce dose-dependent target engagement in healthy volunteers as a subcutaneous sodium-taurocholate co-transporting polypeptide (NTCP) editor. Korro’s KRRO-110 generated a corrected protein in AATD patients but fell short on its efficacy goal—a reminder that the rate-limiting variable in humans is the editing magnitude, not the mechanism.

**Table 1:** ADAR-mediated RNA editing in the clinic and in late preclinical development (as of August 2026)

Program (sponsor)	Target/indication	Delivery	Status and key readout (to August 2026)
WVE-006 (Wave Life Sciences)	*SERPINA1* Z allele; α1-antitrypsin deficiency	GalNAc, subcutaneous	phase 1b/2a RestorAATion-2: first therapeutic RNA editing in humans (2024); updated multidose data (2026) report edited M-AAT at up to ∼60% of total AAT, consistent with the heterozygous genotype with lower risk of disease.
AX-0810 (ProQR; Axiomer—the platform used in this study)	NTCP; cholestatic liver disease	GalNAc, subcutaneous	phase 1 in healthy volunteers: dose-dependent target engagement across key bile acid biomarkers; no serious adverse events or pruritus; plasma half-life ∼8 weeks; first clinical validation of Axiomer.
AX-0811; AX-0422 (ProQR)	NTCP; IDUA (MPS I Hurler)	GalNAc, subcutaneous	clinical trial applications filed or planned; initial healthy volunteer data expected from late 2026.
KRRO-110 (Korro Bio)	*SERPINA1* Z allele; α1-antitrypsin deficiency	LNP, intravenous	phase 1/2a REWRITE: functional M-AAT generated in patients but below the projected threshold after single dosing; program changed to GalNAc delivery.
B4GALT1 program (De Chiara et al., this issue)	*B4GALT1* p.Asn352Ser; atherogenic dyslipidemia	LNP, intravenous (mouse)	preclinical: 9%–18% hepatic editing; −30% galactosyltransferase activity; sustained lipid, fibrinogen, and CETP lowering over 45 days.

AAT, α1-antitrypsin; CETP, cholesteryl ester transfer protein; GalNAc, N-acetylgalactosamine; IDUA, α-L-iduronidase; LNP, lipid nanoparticle; MPS I, mucopolysaccharidosis type I; NTCP, sodium-taurocholate co-transporting polypeptide. Clinical entries are compiled from sponsor disclosures current to August 2026 and are not all peer reviewed.All strategies aim at recruiting endogenous ADAR with a chemically modified oligonucleotide. The enzyme ADAR does not need to be delivered.

The most instructive feature of this study is the amplification of the outcome of editing starting from small initial editing levels. Such amplification is expected when the edited product is a catalytic enzyme acting on many substrates, and it argues for choosing nodal enzymes as editing targets. Modest editing efficiency, the standing criticism of the ADAR platform, may be irrelevant when the downstream biology multiplies it. Genetics also brackets the window: ∼50% loss of activity is usually tolerated, whereas complete loss is often pathogenic; so, the ∼30% reduction achieved here seems appropriate (Figure 1C).

Several caveats remain. The 45-day design measured the metabolic and coagulation endpoints, not plaque—the logical next experiment in a model chosen for atherosclerosis readouts. No washout arm was included; so, the reversibility repeatedly claimed for RNA editing is argued rather than shown. Editing was lower in hyperlipidemic mouse model than in wild-type mice (15% versus 27%), and the transient inflammatory signature requires follow-up under chronic dosing. Finally, intravenous lipid nanoparticle (LNP) delivery is no proposition for lifelong prevention due to safety concerns.^10^ GalNAc conjugation is a potential next step (Table 1), although durable extrahepatic delivery remains to be established.

This study pioneers the installation of an advantageous allele rather than the correction of a defective one, opening a large space for future therapies.

## Acknowledgments

Work in the lab of M.F.J. is funded by the Austrian Science Fund, grant #F80-07 (grant-https://doi.org/10.55776/F80, grant-https://doi.org/10.55776/PIN3964724), and EXCELLENT SCIENCE - Marie Skłodowska-Curie Actions (grant-https://doi.org/10.3030/956810).

## Declaration of interests

M.F.J. has a US and European patent application for the use of edited filamins as a therapeutic agent.
